# Supplementary material for: Stress and Bio-Ethical Issues Perceived by Romanian Healthcare Practitioners in the COVID-19 Era
Source: Int J Environ Res Public Health. 2021 Dec 3;18(23):12749. doi: 10.3390/ijerph182312749 (PMC8657628; doi:10.3390/ijerph182312749)
Supplement: Supplementary file 1 [file ijerph-18-12749-s001.zip › ijerph-1469828-supplementary.pdf]

Supplementary A

We cross-tabulated the activity sector with the years of experience to see the distribution of respondent categories across clinical settings (see Table S1). Similar distributions of practitioners with 10-20 years and 20-30 years of experience (26%) were noted in inpatient and outpatient settings.

Table S1. The distribution of practitioners' medical experience across activity sectors.

Activity sector	Experience					
	0-5	5-10	10-20	20-30	Above 30	Total
Inpatient	10 (20%)	7 (14%)	13 (26%)	13 (26%)	7 (14%)	50
Outpatient	5 (20%)	4 (16%)	7 (28%)	5 (20%)	4 (16%)	25
Administration	1 (25%)	1 (25%)	1 (25%)	1 (25%)	0	4
Management	4 (50%)	0	3 (37.5%)	1 (12.5%)	0	8
Academic	2 (18.2%)	2 (18.2%)	2 (18.2%)	2 (18.2%)	3 (27.3%)	11
Dispensary	0	0	0	1 (25%)	3 (75%)	4
Pharmacy & pharma industry	0	0	1 (16.7%)	4 (66.7%)	1 (16.7%)	6
Private practice	1 (14.3%)	0	4 (57.1%)	1 (14.3%)	1 (14.3%)	7
Other	2 (33.3%)	0	1 (16.7%)	3 (50%)	0	6
Count	22	11	22	27	15	97

Supplementary B

Bio-ethical challenges in the COVID-19 era

This questionnaire contains five sections and will take about 10 minutes to complete. It explores the stress and bio-ethical issues experienced by healthcare practitioners in 2020 during the COVID-19 pandemic. The anonymity of your response is guaranteed. Please answer the following questions.

1. Please check the region of your present activity:
a. Banat
b. Bucharest
c. Dobrogea
d. Moldova
e. Muntenia
f. Oltenia
g. Transylvania

2. Please check the total years of professional experience in the medical field:
a. 0-5
b. 5-10
c. 10-20
d. 20-30
e. above 30

3. Please state your present occupation:

4. Please mention the current activity sector (check all that apply):
a. Outpatient facilities
b. Inpatient facilities
c. Administration
d. Management
e. Academic
f. Maintenance
g. Dispensary
h. Pharmacy and pharmaceutical industry
i. Other (please specify):

5. What is your experience with COVID-19? (Please check all that apply):

a. I had the disease – asymptomatic or with mild symptoms
b. I had the disease – with moderate or severe symptoms
c. One or more family members or close friends had the disease
d. I suffered the loss of a family member or a close friend due to COVID-19
e. I attended patients with COVID-19
f. I had no direct experience with COVID-19
g. I read a lot about the virus, disease, and vaccine
h. Other (please specify):
6. Throughout 2020, were you in the position of making decisions affecting others? (Please check all that apply):
a. Yes – family members
b. Yes – colleagues
c. Yes – subordinates/staff members
d. Yes – patients
e. No
7. Please rate your general wellbeing throughout 2020 on a scale from 0 (very poor) to 100 (very high):
0 100
8. Throughout 2020, how much were you disturbed by any of the following:

	Never	Seldom	Moderate	Often	Always
Irritability, nervousness					
State of concern					
Relaxation difficulties					
Anxiety, unrest					
Anticipation of a negative event occurring					

9. If you were in a leadership position and a pandemic affected the country you governed, how willing would you be to sacrifice individual liberty for the common interest? Please rate your response from 0 (not at all) to 100 (very willing):
0 100
10. Thinking retrospectively, please rate the probability that you would recommend to another state the measures adopted by the Romanian government during the pandemic in 2020?
0 100
11. The following values are important to me (Please rank the values in the order of their importance, starting with 1 = most important):
Wisdom
Safety
Love
Freedom
Health
Happiness
Honesty
Faith in God
Wealth
Empathy
Living without sin
12. In 2020, mass media information (TV, radio, and written press) was:
a. Useful
b. Exaggerated
c. Insufficient
d. False
e. True
f. Helpful for overcoming this period
g. Not good for me
h. Confusing for me

13. Which of the following fundamental bio-ethical principles do you think were infringed on a social level during the SARS-COV-2 pandemic in 2020? (Please check all that apply):
a. Beneficence (the imperative of doing good)
b. Nonmaleficence (the imperative of not doing harm)
c. Autonomy (the imperative of respecting patients' capacity to decide for themselves)
d. Justice (the imperative of correct resource distribution)
e. Truth (the imperative of transmitting accurate medical information)
f. None of the above
14. Which of the following bio-ethical principles did you personally infringe? (Please check all that apply):
a. Beneficence (the imperative of doing good)
b. Nonmaleficence (the imperative of not doing harm)
c. Autonomy (the imperative of respecting patients' capacity to decide for themselves)
d. Justice (the imperative of correct resource distribution)
e. Truth (the imperative of transmitting accurate medical information)
f. None of the above
15. Which of the following principles would you find the hardest to infringe?
a. Beneficence (the imperative of doing good)
b. Nonmaleficence (the imperative of not harming)
c. Autonomy (the imperative of respecting patients' capacity to decide for themselves)
d. Justice (the imperative of correct resource distribution)
e. Truth (the imperative of transmitting accurate medical information)
16. If you were in a leadership position, in what order would you recommend vaccinating the population against COVID-19? (Please rate the following categories in the order of importance, from 1 = most important):
Old and sick people
Women and children
Government officials
Military personnel
HORECA personnel
Healthcare workers
Educational workers
Pupils and students
Industry personnel
No one
17. Please describe the most complicated bio-ethical decision you had to make in 2020:

18. Please check all the situations you have personally encountered throughout 2020:
a. Infringing patients' rights (e.g., the right to informed consent, confidentiality)
b. Hospital admission without the patient's consent
c. Hospital discharge of COVID-positive patients upon request
d. Hospital discharge of a patient with delirium upon request
e. Limiting access of patients' families to the hospital
f. Neglecting non-COVID-19 patients
g. Neglecting medical assistance of patients with chronic illnesses
h. Triage of patients (selecting patients to have access to intensive care)
i. Choosing between self-protection and compliance with professional duties
j. Lack of intensive care technical utilities that led to triaging or redirecting patients to other medical centers
k. Pollution with single-use materials
l. Obtaining informed consent in the correct way (cultural limitations)
m. Administering the mandatory treatment for COVID-19 (with seemingly appropriate medication)
n. "Blind" application of protocols
o. Refusal to treat COVID-19 patients
p. Restricting access of non-COVID-19 patients to medical services and medication

r. The stigma associated with COVID-19
s. Managing hospital sections at surge capacity
t. The conflict between personal beliefs (e.g., opinion on the vaccine) and the professional line of conduct
u. Reluctance to express personal opinions as a healthcare worker
v. No such situation

Thank you for your participation!
